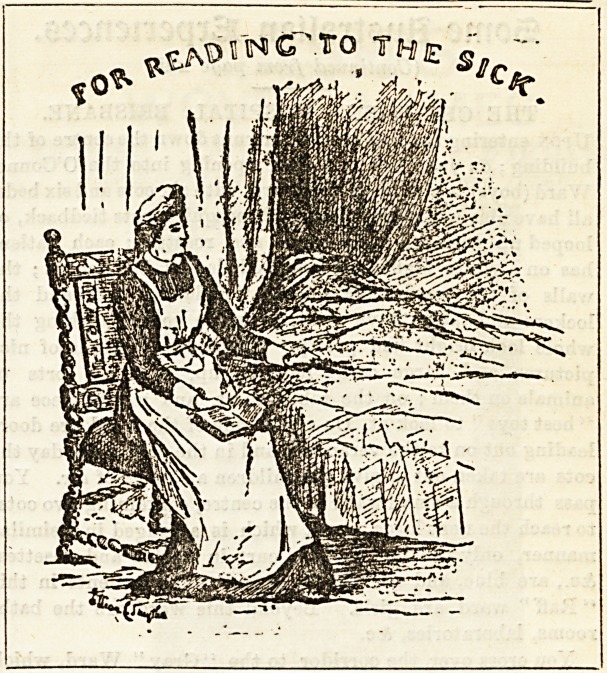# The Hospital Nursing Supplement

**Published:** 1892-12-10

**Authors:** 


### "The Hospital" Nursing Mirror.

Being the Extra Nursing Supplement of "The Hospital" Newspaper.

[Contributions for this Supplement should be addressed to the Editor, The Hospital, 140, Strand, London, W.C., and should have the word "Nursing" plainly written in left-hand top corner of the envelope.]

### En Passant.

THE PRIVY COUNCIL AND NURSE REGISTRATION.—The date when the Privy Council will give its decision has not yet been fixed, but the case will be put into the paper and the judgment announced in the Council Chamber.

FUNERAL HONOURS.—It has been resolved by the Paris Municipal Council that any nurse, of either sex, who dies in a hospital of disease contracted in the discharge of her duties shall be buried in ground assigned in perpetuity, and in future, when a nurse dies under these circumstances, the Municipal Council will send a delegate to represent it.

OUR CHRISTMAS PARCELS.—Will any of our readers who can help us this week collect everything they can in the shape of adult clothing for distribution in the hospitals. If every friend of The Hospital will send us something this week or next, and we can receive parcels up to Thursday, 15th, we shall have no difficulty in getting all we know is needed. Out of all our great number of correspondents there are many who can afford to do a very little, and we do earnestly ask for extra help this year, all of which we will guarantee shall be sent where it will be used to the very best advantage.

A NURSE FOR SEAHAM HARBOUR.—There has certainly been no more consistent friend to nurses than the Marchioness of Londonderry, she has befriended them wherever she has been, and once more she is going to help start district nursing, this time for Seaham Harbour, and has promised substantial aid in the shape of funds from herself and Lord Londonderry to support the expense of one nurse. Like the Stockton Association, which is a complete success, and of which Lady Londonderry is President, the various collieries and works will be invited to send representatives to the committee; this has in the case we mention been a very great help to the finances, the working men's subscriptions providing a very large part of the working funds.

WOMEN'S CONVALESCENT HOME ASSOCIATION—At the usual monthly meeting of the Ladies' Committee of the above Association, which numbers amongst its workers Lady Constance Howard and Lady Henry Somerset, held at the Memorial Hall, Farringdon Street, the Secretary (Mr. R. Frewer) reported that 99 women and girls were passed through the Home at Great Wakering during the first four months, from July to October inclusive, and in every case with very beneficial results. Many of the residents had been teachers or lady clerks, who spoke enthusiastically of the rest and change afforded, and of the high character of the Home. The total expenditure had been £515; the total contributions about £300. The following letter was read from Mr. Howard Morley: "Dear Sir,—I have heard a good account of the beginning you have made; and in the hope that the Women's Convalescent Home movement may make rapid progress, I gladly send you the enclosed cheque for £50 in aid of its funds." Amongst other amounts received was £5 5s. from Messrs. Bryant and May. The Home will at present accommodate about 500 visitors each year, but the committee are hoping to be able to increase the number of beds in the spring, it being expected that many employers and mistresses will avail themselves of the opportunity afforded of giving those dependent upon them the benefits of rest and change in cases of overwork or recovery from illness. Applications for admission should be addressed to the Secretary, 29, Memorial Hall, Farringdon Street.

GLASGOW SICK POOR AND PRIVATE NURSING ASSOCIATION.—The third annual meeting of this association, founded by Mary Orrell Higginbottom, took place at the Home in Bath Street, and was well attended. There are now sixty-four nurses on the staff, exclusive of the home and superintendents of the districts; thirty-nine of these are private nurses, fifteen are doing district work, and ten are probationers in training. Since last year the working hours of the nurses have been increased one hour a day, in order to correspond with the hours of the Q.V.J.I.N.; the nurses, we are glad to say, forbore parading the streets with a banner in order to protest against this addition to their labours, and are cheerfully working away, gaining gratitude and confidence from dwellers in the poorest parts of Glasgow. Proud as the city seems of the achievements of its nurses, it has not yet provided enough good men and true to relieve the association of all financial anxieties.

SHORT ITEMS.—Miss E. A. Wickham, on resigning her post of Night Superintendent at Homerton, was presented by the night nurses with a beautiful hypodermic syringe and Heath's "Minor Surgery and Bandaging."—A sale of work will be held on December 20th in aid of the St. John's Nursing Home, West Kirby, Cheshire, at the Home. —On Monday and Tuesday, January 2nd and 3rd, a sale of work in aid of "Friedenheim," the Home for the Dying, will be held at the Hampstead Conservatoire, and any help will be gratefully received by Miss Davidson, at the Home.—Cinderella dances in aid of the Popular Musical Union will be held in Chelsea Town Hall on January 17th and February 7th under the management of Mrs. Garson, Mrs. Ernest Hart, and others. Application for tickets must be made to the Secretary of the Union, 33, Brewer Street, W.—Parcels received with many thanks from Miss Chisnall and Nurse Eliza Smith, and some nice knitted cuffs from Miss Neal.—Gateshead is to have a self-supporting nursing association, and we hear that a similar undertaking is being meditated at Colchester.

BELFAST NURSES' HOME AND TRAINING SCHOOL.—The twenty-first annual meeting of this home was presided over by Lady Shaftesbury, who is a very staunch and kind friend both to the Royal Hospital and the Nursing Home. There are now eighty-four nurses and probationers, but during the year there has been quite an exodus, happily, however, a pleasant one, of some of the best nurses on the staff. One nurse left to become matron of the Hospital for Consumption, Belfast; one to be nurse matron of the Queen Victoria Jubilee Convalescent Cottage, Belfast; one to be head nurse of the Charitable Institution, Belfast; one to be charge nurse at the Protestant Infirmary, North Dublin Union; one to be night nurse at the Cumberland Infirmary, Carlisle; one to join the private nursing staff of the West Kent General Hospital; three to be district nurses —two in Belfast, one in Larne; one to take private nursing on her own account; and one left to be married. We are sorry to hear that there is still an adverse balance in the accounts. Belfast ought to support the Home, for the Royal Hospital, which is entirely worked by these nurses, has gone up in popularity by leaps and bounds, and also the work is not by any means confined to taking paying cases. The district nursing is all worked by the Home, and Miss Newman has also been sending out a nurse on short visits to those who can afford a very small fee for daily nursing.

### Lectures for Asylum Attendants.

By William Harding, M.B.

(*Concluded.*)

### X.—ENTERTAINMENTS.

To superintend the amusements of the patients becomes after a time the hardest part of one's duties. It requires an immense amount of perseverance to carry on such entertainments year after year without getting wearied and listless about the business. If, however, the interest of the staff in these matters is allowed to flag, they speedily degenerate and become flat, stale, and unprofitable. We must remember to suit the style of our entertainments to the people whom we wish to amuse. To send a party of patients to a per-performance which they are unable to appreciate, and from which they return bored and disinclined to attend another, is worse than a mistake. There is something very wrong when strong moral suasion has to be used to induce patients to go to the hall. Some of our professional entertainers appear to forget that to the lunatic, as well as to the sane individual, repetitions of the same performance are not likely to call forth much enthusiasm. Probably the most generally popular of all entertainments are the dances. There is lively music, with dancing, for the active ; and when discipline is not too rigidly maintained, there are opportunities for gossip for those who are past such exercises. In some asylums the nurses are allowed to dance with the female patients ; in others this is forbidden. When there are private dances in the female wards for women only the objections to the latter system are greatly overcome. It often happens that it is in the very cases which would be taken up to dance by the nurses, but would not be likely to dance with males, that we hope for most benefit from the exercise. Each asylum has it s own traditions in such matters, and undoubtedly each has its good points. There is always a danger that we hold too closely to the old form after the life and spirit have fled. Concerts and coffee parties are other favourite entertainments. The latter are very successful when cheerful and lively, but are most depressing when the patients cannot break through the bonds of routine and enjoy themselves heartily and rationally. A subdued whispering murmur with no individual daring to make his or her voice ring out clearly is a fatal sign. Patients will never thoroughly enjoy themselves until the staff are sufficiently interested to start the entertainment off with a swing. Fancy dress balls are held in some asylums. The great advantage of these is that for weeks before the ball comes off, and after it is over, there is something to talk and think about. Cricket matches in summer practically take the place of theatrical entertainments. In addition to the benefit derived by those who play during the week, the regular match provides a weekly spectacle and subject for conversation.

The entertainments which are most successful are those given by individuals known to the patients, and more especially if the performers are those with whom they are intimately associated. In such cases a high degree of excellence is not demanded. Anything done in this way with a desire to interest and amuse, by nurses or attendants, is more appreciated than performances given by expensive professionals.

We want more social life amongst the insane. There might with advantage be more mingling of the sexes under, of course, due restrictions and precautions. Men and women who are quite capable of associating with their fellow creatures, and of benefiting by such society have the social side of their life slowly starved to death. Just picture the daily life of many a female patient. She plods the same eternal round : she gets up, eats, works, and goes to bed ; sees no one but her fellows, and is continually subject to their squabbles and petty annoyances. Life in a nunnery is a rollicking picnic to such an existence. Men, too, spend their years in tramping from sunrise to sunset in the same old rut, which only gets deeper day by day, while they grow more and more demented. This is simply creating a species of asylum dementia, and doing it in those cases which might with least advantage be hurried into that living death. We want more cheerfulness in some of our patient's gatherings—a greater sense of freedom and absence of formality. In large gatherings, as in dance halls, this is very difficult, but there is no reason why selected parties of men and women should not meet together, and mingle freely with mutual advantage. There are, of course, many who could not be admitted to such gatherings. Still it is wonderful how self-control develops when there is something which is worth the exercise of that quality. Social evenings of this kind imply constant watchfulness, and necessitate a very accurate knowledge of the patients amongst whom you are living. It is in circumstances such as these that one appreciates nurses and attendants who have intelligently observed those under their care. Though every patient in such a meeting is under observation, such a fact need not be made apparent. Men and women from the same town can meet and discuss acquaintances ; they can play cards, draughts, chess, or they can listen to the music as they please. Small carpet dances under such circumstances are generally successful. In the better wards the female patients may occasionally invite those of their acquaintance amongst the men to tea, after which are dances, games, &c.

As a rule in county asylums few patients are found who can assist in theatricals. These, almost invariably, are left to the staff. An effort should be made to render one of the entertainments the peculiar property of the patients, that is, to make it an entertainment by patients for patients. Such a weekly evening's amusement has been carried on for years in one asylum. The patients themselves sing, play, and recite, and they ask members of the staff to assist by invitation. Each programme is submitted beforehand to the medical officer. He strikes off the name of any individual who is not thought then in a fit state to take an active part. The doctor, with, of course, nurses and attendants, is present, but takes no part in the proceedings unless anything unusual occurs which calls for his intervention. The chairman of the concert presides and manages the business. In this case the concert was held in one of the male day rooms and female patients went to it regularly. It was one of the most popular entertainments in an asylum where amusements were rather a specialty. Anything in which all can take a part is much appreciated ; hence the popularity of songs with a good chorus. This, combined with the lively airs, is the reason why the singing of Sankey's hymns on a Sunday evening is always so much looked forward to.

Entertainments are employed as curative agents, and also to lighten the lot of those unfortunate people who are condemned to live apart from their fellows. We endeavour by such gatherings to civilise and build up again a little self-control. I would rather see a few turbulent patients removed from a meeting, than have the chairs filled with utter dements, safe to give no trouble, but past salvation, and quite beyond being influenced either one way or the other. Entertainments are not to be used indiscriminately. I have seen harm done by allowing cases in the first stage of convalescence from acute mania to attend such things too early. On the other hand, I have known very marked benefit result, and I have seen cases where individuals appeared to be sinking into dementia, and, as it were, dragged back by the revival of their social instincts.

Let us by all means try to give the patients something to think about ; to occupy their minds to the exclusion of their individual worries, and the everlasting discharge day. If even for only a couple of hours in an evening, we can make them forget that they are confined within the walls of an asylum, our time and trouble will not have been thrown away.

### Merit and Modesty.

### A NURSE'S VIEW.

Yes, personally I certainly do like most congresses. They are amusing and occasionally instructive and give us opportunities for hearing other people's views on many subjects. Even if not altogether of a practical nature, some of the theories enunciated give food for thought, which is doubtless good for nurses w ho are popularly supposed to have narrow ideas and to be limited in the matter of intellect! A great many presumptions spring from a grain of truth and so it is never safe to condemn such suggestions as entirely unfounded. Of course, some of us are narrow, but so are other women who have less excuse for neglecting the cultivation of their minds. However, real working nurses are generally distinguished, albeit in a quiet, humble way, for unwearied devotion to their profession. Matrons, sisters, charge nurses, in fact all persons thoroughly trained to take intelligent care of the sick, are intent on doing this quietly, as well as faithfully. To our own nursing schools only do we each owe a debt which can be paid fully by creditable subsequent careers. Thus alone do we bear witness to the efficient and complete training which has equipped us for our fight against disease and death, ignorance and prejudice.

The varied experience which each must personally acquire, should tend to the ultimate evolution of that ideal nurse, the typical woman formulated by the immortal example and consistent teaching of the great Florence Nightingale.

Said a clever and quiet-looking middle-aged nurse the other day, "How I wish that people would just let us alone, treat us to a little wholesome neglect, in fact! Of all work attempted by women, surely ours should be altogether tranquil, and certainly unemotional. How can we remain modest and gentle and unselfish now that we are forced upon public notice, in season and out of season? We get talked about and written about and popped on to platforms. We are also introduced into sermons and dragged into pamphlets. We are photographed and sketched, and our uniform is freely discussed, not with regard to its utility or suitability, but with respect to its becomingness. The subject of our earnings has become a burning question of the day, whilst folks who have never employed a trained nurse in their lives feel quite competent to lay down the law respecting the exact kind of woman best suited to the work."

Oh! kindly general public, please leave us to ourselves a little while. There is doubtless a section of the nursing profession which loves notice and clamour, but that is not the class of women who bring comfort into sick chambers. When really ill, you want quiet and unobtrusive service, and you won't be able to secure this from any body soon, if you encourage romantic sensationalism.

We shall learn very quickly to pose for the public, some of us are already inclined to do so, and a few bad examples find many weak-minded imitators.

The flood of forced excitement seems indeed threatening to overwhelm quiet folks, for a Congress of Nurses is actually talked of at the Chicago Exhibition. Surely the title is amazingly discordant, and is little likely to find favour in good schools of nursing in the New or the Old World. Sober Englishwomen will never consent to neglect the plain claims of their noble calling for the sake of attending mass meetings. Idle projects can never displace those honourable duties which the pioneers of nursing have laid down so calmly and reasonably for their followers.

It is not the tried and worthy English and American nurses who need a "conference" to extol their virtues; the sick and the sorrowful are well acquainted with the value of such women as these. As for the incapable or sensational members of the profession, they may possibly need the hall-mark of publicity to ensure for them some sort of general recognition.

Those of us who have time and money at our disposal would assuredly derive much advantage and mental refreshment from a journey to Chicago, but whenever we nurses go to visit our American neighbours let it be as observant spectators only.

### Notes on Novelties.

### NURSES' UNIFORMS.

We have often occasion for remarks upon dress, although at first sight it does not seem a very essential subject for such a professional paper as The Hospital to take up. But on second thoughts most people will own that a nurse's costume is important and significant. We dare not say that every properly dressed nurse is a good worker, but we can safely assert that untidy or unsuitable garments are often an index to a character. Much depends, of course, upon how things are put on, but there is a better chance of this being satisfactorily accomplished when each article is perfect in itself. A neat bonnet means one that fits the wearer, of a suitable size, and a becoming shape; it should be distinctly useful when it is worn by a nurse, not a mere ornamental head-dress. We advise probationers to give full consideration to the subject of uniform when they first take to it, for a little care as to shape and fit of garments when they are chosen will enable any average woman to escape all troubles later on with regard to wearing her clothes. They will seem merely part of herself, and she will have her mind free for other points connected with her training. We are very much struck by the excellent style of things provided in Messrs. Debenham and Freebody's special department. They have so many pretty shapes for nurses' cloaks, "The Cavendish" being a particularly pretty and becoming one. Made in a soft, light, and very warm cloth, specially manufactured for the purpose, and warranted shower-proof, it suites the requirements of hospital, private, or district nurse. The bonnets at this establishment are well worth inspection by any nurse, for they are remarkably cheap as well as pretty and neat. Materials for washing dresses are many and varied and suited to every kind of taste. A really well-fitting cloak, whether it be a "Russian," "Cavendish," "Wigmore," or any other, is certain to prove satisfactory, and as we said before, nicely-shaped garments are essentials in "uniform" costumes. We congratulate Messrs. Debenham and Freebody on the success with which they have provided for all the reasonable requirements of the nursing world at their establishment in Wigmore Street.

### A New Departure.

One of the many useful bits of work carried out by the Metropolitan and National Nursing Association is sending a nurse to the Board schools to see to all minor ailments. There is an interesting account of it in "Nursing Notes" for December. She sees all children with "blight" (catarrhal ophthalmia) unhappily very prevalent, discharging ears, sore and dirty heads, abscesses, small burns, broken chilblains, &c., all minor ailments which are made the excuse for detaining children from school. In the milder forms of ophthalmia it is found sufficient to thoroughly cleanse the eyes with warm boracic lotion—this cures them very quickly. If severely affected, the nurse goes to the child's home, and if necessary insists on its attending either a hospital or dispensary, and she carries out the orders given. The same rule applies to ear mischief, ringworm, and other definite diseases, which the parents are too indolent or ignorant to attempt to treat. This experiment has proved successful so far, both as regards the attendance of the children at the schools, and also in enabling the nurse to detect real disease of eyes, ears, or skin, and thus getting the child under proper medical treatment.

### Everybody's Opinion.

*[Correspondence on all subjects is invited, but we cannot in any way be responsible for the opinions expressed by our correspondents. No communications can be entertained if the name and address of the correspondent is not given, or unless one side of the paper only be written on.]*

### THE DAILY WALK.

"Ignoramus" would like to know if a nurse can demand an hour's walk daily? Some time ago a sick lady required the services of a trained nurse, and when the engagement was made, the nurse was told she should go out as often as convenient. In nine days she was out six times, but was impertinent because she was kept in when the patient had a rising temperature, or when it was not convenient for anyone else to take charge of the patient. When told the patient ought to be the first object, she said there was "no fun" in nursing unless she got what she could properly demand—an hour's walk a day, convenient or not.

"R. C." writes in answer to the annotation on this subject, which appeared in our columns last week, and which was apropos of the letter from "Ignoramus" printed above, which was unavoidably held over owing to pressure of space: In reference to an annotation which I saw in The Hospital of 3rd inst., I was under the impression that nurses had two hours off duty each day. As to convenience, I think a nurse is the best judge as to when her patients can be left, and I have usually found someone in the family able and ready to look after my patients when I have thought they could be left with safety. Perhaps I am more fortunate than some of my fellow workers with my patients, but I think they are often what their nurses make them; and it is always necessary to hear both sides of a story. Nurses ought to go out every day, but there are few who care to leave their patients for the few days when they need them most. It is only fair, however, that time lost in this way should be made up when the patient is better, and I think most patients will agree in that. There are some patients who think their nurses are machines; they forget that a nurse has probably been working several years, and will still, probably, have to work for a few more, and that she cannot afford to give her strength by constantly attending her patient day and night, as an ordinary friend would do, for a friend is generally able to rest after a strain of nursing. Such patients should have the rules given to them. I think most nurses are willing to give as much time to their patients as is necessary, but we all know that a certain amount of fresh air and rest is essential for every nurse who is expected to do her duty well, and invalids are apt to grow selfish without being aware of it.

### ST. JOHN'S MATERNITY HOME.

Miss Ritchie, Hon. Sec. of the Clapham Maternity Hospital, writes: Seeing a notice in The Hospital of November 26th respecting the closing of St. John's Maternity Home at Battersea, I venture to ask you to let me explain in your next issue that although the Maternity Home in question is no longer under the charge of the St. John's Nursing Association, it is a mistake to speak of it as closed. Its work was by special arrangement with the Nursing Association taken over by the Clapham Maternity Hospital, and St. John's House, Albert Road, Battersea, still remains the centre of active out-patient work of about 650 cases per annum. This work was carried on in full swing by the St. John's Nurses till the evening of October 31st, when they were replaced by the new workers from Clapham, including two resident (lady) medical officers and a certificated midwife, besides the staff of nursing students, and there was thus no break whatever in the continuity of work. It is true, however, that St. John's House, Battersea, is closed as a "maternity home." No in-patients are now received there, all such cases having been since November 1st received instead at the Clapham Maternity Hospital, 41, Jeffreys Road, eighteen minutes' drive from St. John's House, Battersea. It is thus hoped that the excellent work begun and carried on for so long by the St. John's Nursing Association has by no means come to a close, but will continue a healthy existence under a different name.

### The Nurses' Bookshelf.

### PILCHER'S FIRST AID IN ILLNESS AND INJURY.*

This book is graphically described on its title page as a series of chapters on "The Human Machine." The author is Dr. James Pilcher, captain in the medical department of the United States Army, and he has sought to produce a text-book for civilian and military first aid classes as well as a manual for quick references in the emergencies which arise before everybody in their turn all over the world. At first sight the manual appears, perhaps, too copious for ready use, but closer investigation will show that a distinction has been made between essential points, and details which might be omitted by using large type for primary and important facts, and by keeping the minor facts entirely to the smaller type.

The first part contains a clear understandable anatomy and excellent chapters on the organs and senses; part two is headed the "Implements of Repair," and contains the theory of germs, their action and control, the use of knots and bandages, and the various dressings and applications. In the chapter on "Knots," the "granny" knot dear to most of us as the elementary attempt at knots of our childhood is ruthlessly yet amusingly illustrated as what "should not be tied." Part three, on accidents and emergencies, strikes us as being exceptionally good; the directions as to how to examine an injured person have all the care and thoroughness prompted only by experience, the points to be observed in the treatment of all accidents are explained clearly and concisely, and the further details given on each subject in the smaller type will, in our opinion, assist very materially to the more intelligent treatment of sick and wounded who may find themselves at any time stranded far away from the ministrations of doctor or nurse. Chapter twenty-seven is entirely devoted to the emergencies of the battle-field, the improvising of litters, litter drill, and many other points of like importance. The last part of this excellent handbook shows how to take care of "The Human Machine" by providing proper ventilation and sanitary precautions in our dwellings, and by using our food with some consideration of its necessary elements.

### MOTHER'S CARD FOR REARING INFANTS.†

This curious title appears on a card which has been forwarded to us, and the facts herein set forth are so confused that they will probably puzzle the mothers it aspires to educate. Under the heading of "Infants' Food," we find "Windows should be opened at least three times a day for a few minutes; if cold weather five or ten minutes will do." It would be instructive to find out what this "will do" means—not nourishment, and surely not efficient ventilation! The quantities of the food, and the intervals between its administration are far too vaguely treated for acting upon. But the worst feature of the card "for rearing infants" lies in its prescriptions. Doctors are aware from sad experience of the propensity of the uneducated classes to take physic. They love doses of medicine, and the intelligent country practitioner wages incessant war against the dangerous habit of drug-taking without medical authority for it. Here we find diarrhœa, constipation, feverish symptoms, sore throat, teething, and thrush, are all to be treated with mercury and chalk powders at the mother's discretion or indiscretion. To conclude with, we are told that permanganate of potash is a disinfectant and antiseptic, and that one or two crystals dissolved in half-a-pint of water, make the requisite strength. Such "disinfection" as would be accomplished by these means would hardly prove efficacious, we imagine, in the matter of contagious diseases!

* "First Aid in Illness and Injury." By Dr. James Pilcher. (Kegan Paul, Trench, Trubner, and Co.)

† "Mother's Card for Rearing Infants." R. S. Doxford, 42, Evesham Street, Redditch.

### Examination Questions.

### ON CERTAIN POINTS IN THE NURSING OF TYPHOID.

The best answer sent in was sent by Nurse Agnes, West Ham Hospital, Stratford, E., and is printed below. We received seventy answers to the November question, varying very much in thoroughness, but they were on the whole satisfactory, and we were especially glad to find such a number of nurses ready to send up papers. There was rather a remarkable similarity in some of the answers, and we think the suggestion made by a correspondent in our columns for November 26th is the best way out of a difficulty. Our questions are meant to incite nurses to take an intelligent interest in their cases, and to prevent knowledge from getting rusty. So for the future will nurses kindly answer from their own experience and knowledge. There will not be much time for writing during the next three weeks, but a short question can be answered without much arduous work. That for December is: "You are nursing a patient with rheumatic fever; the doctor has paid his daily visit and lives miles away. In the afternoon you find the sweating has ceased, there is slight delirium, and the temperature is 106 deg.; what would you do?" Answers must be sent in by December 31st. They must be accompanied by writer's name and address, must be as concise as is compatible with the subject on which they are written, and must be written on one side of the paper only, and addressed "Nursing," Editor of The Hospital.

### Prize Answer.

The ileum, or third portion of the small intestine, is the chief part attacked by the typhoid poison. Here are situated glands called Peyers glands or patches, and the nature of the disease consists in inflammation and ulceration of these. During the second or third week of the disease the ulcers slough, leaving this part of the intestine very thin and delicate. Hence the importance of keeping typhoid patients absolutely at rest in the recumbent position, as any movement, such as suddenly sitting up, or standing, might rupture these delicate parts, causing perforation, and ending almost certainly in fatal peritonitis. Another complication of typhoid fever is hæmorrhage from the bowels. This occurring slightly within the first fortnight of the disease need not give rise to great anxiety, but after that time is a symptom of grave importance, and medical advice should be at once procured. The nurse should be able to tell at once when internal hæmorrhage is going on, as the symptoms are well marked. The patient becomes pale and giddy, and breathes with difficulty, a cold perspiration breaks over the body, the pulse becomes slow and feeble, and the temperature falls suddenly, generally to sub-normal.

### Death in our Ranks.

It is with feelings of deep regret that we record the death of Nurse Dickson, of the Central Corporation of Nurses, Edinburgh, who expired in Leith Hospital on Friday, November 25th. Nurse Dickson voluntarily nursed a sick patient suffering from typhus fever and caught the infection, her strength having been much over-taxed in a private case.

### Notes and Queries.

### Queries.

(33) *Australian Experiences.*—Can any correspondent tell me in which number of The Hospital there is an article headed "Some Australian Experiences"?—*Petronella.*

### Answers.

(33) *Australian Experiences* (*Petronella*).—The Australian Experiences have appeared in the numbers of June 18th, August 27th, September 3rd, October 15th, October 29th, November 12th, and they will be continued as regularly as possible.

### BITTERNESS.

There is an affliction which befals many people whether they are ill or well, but it is most harassing to those who are suffering from a long and tedious illness. It is that weariness and distress of heart which takes all happiness out of us. We are sullen and sad, we dislike the place we are in, and loathe whatever we have to do, while we nurse a poor and sorrowful opinion of our fellow creatures. As a great and good man said, speaking from his own experience, "We turn over a huge heap of blessings in our minds to find two or three fancied evils buried among them." Another poet, Archbishop Trench, sings of

"Long and weary days,
Full of rebellious askings, for what end,
And by what power, without our own consent,
* * * * *
We were placed here to suffer and to sing,
To be in misery, and know not why."

and then goes on to say our lives, at such miserable seasons, seem but as an arrow flying in the dark without any aim. Such thoughts in our hours of weakness cause us to lose all faith and trust in a loving God; but Lord Tennyson, in "The Two Voices," tells how the battle with "crazy sorrow" is to be fought out and the victory gained, "the bitter sullen voice be gone." In all ages men have suffered in like manner. The tempter is always busy whispering rebellion, against truth and light and love, in our ears. In the court, the camp, the hermit's cell, and the hospital ward are his assaults made, and, unable to answer him off-hand, we sink into a sleepy apathy nor strive to shake off the gloom. It is curious to notice how human nature resembles itself at all times and in all sorts and conditions of men. They were as much beset in the fourteenth century as in the nineteenth by these miserable thoughts. And how did they fight then against the adversary? Just as they do now, if they were brave. All that was done then, all that can be done now, is to go on steadfastly working hard or bearing our sufferings with patience and fortitude accordingly as our condition admits, with a strong determination to do our duty. If we are sunk in such sullen gloom and can never turn a bright and cheerful face on our companions, let us say, with Mr. R. L. Stevenson in his noble poem, "The Celestial Surgeon"—

"Lord, Thy most pointed pleasure take,
And stab my spirit broad awake;
Or, Lord, if too obdurate I,
Choose Thou, before that spirit die,
A piercing pain, a killing sin,
And to my dead heart run them in."

It's the wounds of God's love which alone can soften our stony hearts. Its sure cure is love, joy, peace—the fruits of the Spirit; but these cannot live in the heart which deliberately yields itself up to despondency. We must make common cause with our Deliverer, or perish.

### Some Australian Experiences.

(*Continued from page* xlii.

### THE CHILDREN'S HOSPITAL, BRISBANE.

Upon entering the hall a corridor runs down the centre of the building; on your left is a door opening into the O'Connel Ward (boys), a nice large airy ward, with six cots and six beds; all have nice white coverlets, and mosquito nets tiedback, or looped up with red braid bands and rosettes; each patient has on a white nightdress and red flannel bed jacket; the walls of the ward have a deep "dado" of red, and the lockers and tables have red and white covers, making the whole look bright and pretty. There are all kinds of nice pictures and large cards hanging up, with all sorts of animals on them; on the table, chest, and mantelpiece are "best toys" to look at. On either side of the ward are doors leading out on to the verandah, and in the cool of the day the cots are taken out to give the children a change of air. You pass through a small ward in the centre, containing two cots, to reach the next large ward, which is arranged in a similar manner, only the bed jackets, curtain braids and rosettes, &c., are blue, and so is the "dado." The patients in this "Raff" ward are girls. Beyond this ward are the bath-rooms, laboratories, &c.

You cross over the corridor to the "Gray" Ward, which has accommodation for ten patients, generally convalescent. The next along the corridor, as I have said, is the Lady Superintendent's bed-room; and farther on a nice large dining-room, used for both patients and staff. Then came the sitting-room of the Lady Superintendent, and over a partition, the dispensary; this brings us back to the hall again.

The lower part of the building contained nurses' bed-rooms, which were very comfortable; servants' bedrooms, kitchen, an accident ward, containing six cots and beds, arranged in a similar manner to the large upstairs wards, and an eye ward of eight beds and cots, and a nurses' room. We very frequently had to squeeze ten into this ward, but as none were in bed during the day it did not so much matter.

The committee-room, at the back of which was the night nurses' bed-room, finished the lower portion of our "Noah's Ark."

This was a nice large room, and was open for the nurses' use as a sitting-room, but the climate is so fine they generally go out when off duty.

At the lower end of the grounds were the infectious wards, a very compact set of nice little wards, with kitchen, bath-rooms, and so on, and accommodation for night and day nurses, enabling the place to be entirely apart from the main building.

Upon emergency we could accommodate eight infectious patients, but the average was five or six, chiefly diphtheria, scarlatina, chicken pox, and mumps; typhoid was nursed in the medical wards.

The late respected Sir Anthony Musgrave was then Governor, and Lady Musgrave was the Patroness of the Children's Hospital, and thanks to her energy and liberality a very nice sanatorium was opened at Sandgate, a seaside place about fourteen miles from Brisbane. The railway company gave free passes for the nurses and children. Lady Musgrave furnished the "Musgrave" Ward, Lady O'Connel the "O'Connel" Ward. The Lady Superintendent and the Ward "Sister" visited the sanatorium weekly, alternately, and a nurse from the hospital took charge a month at a time. In this way the whole of the staff got a change, as well as the patients, and the whole thing was a great boon.

I cannot speak too highly of the liberality of the Brisbane public; their interesting, useful, and substantial gifts were most thoroughly appreciated. Not only at Christmas, but all the year round cakes, fruit, toys, clothing, linen (new ready-made), bed jackets (boys' and girls'), in fact everything poured in. Biscuits and sweets could be had by sending a line to Mr. Wilson (biscuit factory), or to Mr. Cohen (confectioner), while flowers from the Sunday-schools, festivals, &c., were sent in large washing baskets full.

At Christmas a lovely tree is provided by a gentleman who brings it himself, dresses it, and lights it up, and then gives a most delightful explanation of Christmas to the children before he distributes the prizes. This gentleman does not like his name mentioned. I had the pleasure of being there two Christmases to receive him and I really do not know who enjoyed it most, he or the children.

On the Queen's birthday all the Sunday-schools meet either in Victoria Park or the exhibition grounds, and I was always allowed to send as many of the convalescent children as I could, in charge of a nurse, of course, and they were kindly seen to, too.

In Brisbane I added to my number of kind friends, and real friends, too, they proved at a time when I needed some badly, for I did not go through all my Colonial experiences without trouble, and I shall ever remember with gratitude all who were so kind then.

There was an honorary staff of five doctors to the hospital, who attended daily. I also had most kind neighbours in the General Hospital, just below us on the same road, who were ever ready to render assistance.

The Children's Hospital is very prettily situated on a hill, which, of course, is nice for the hospital, but not for the poor "mothers," who used to arrive quite exhausted after the climb on a hot day, often carrying an infant, and perhaps another child hanging on to their skirts.

The staff consisted of Lady Superintendent, the Ward Sister, and fourteen nurses, five servants, and a little girl I took from the Orphanage, my own special "protégée," and a very good child she proved, too.

I arranged the "off duty" time precisely the same as at "The Prince Alfred," and found it answered very well.

The Brisbane General Hospital is a fine brick and stone building, and through the able administration of Dr. Sandford Jackson and Miss Weedon has become a very popular place. The grounds are very nicely laid out, a great pleasure to the patients; they also have a sanatorium at Sandgate.

There is a nice new lying-in hospital, the "Lady Bowen," which is a great improvement on the old one, and answers splendidly. There is the usual "Benevolent Asylum" a little way out of town, for old folks and incurables.

There was a talk of a South Brisbane Hospital, the sooner it takes some shape the better; it is a long way to bring sick people from south to north as at present.

Brisbane is not half a bad sort of place to live in, but it gets monotonous; the theatres are fairly good, but the best companies only come up there about once in two years; the gardens are pretty; there are also some pretty rides round about, but nothing compares with Sydney, which I fear spoils one. Of course, it is so much younger than Sydney, so one should not draw comparisons.

There is a lovely place called "Loowoomba" on the Darling Downs, which is the country trip, "a run up the range," as it is called. I went up for a few days' rest, and enjoyed it. The hospital there is a good one, in fact, as I have already said, all the country hospitals are nice, but this is exceptionally so; a "Prince Alfred" Sister is in charge, and four nurses. The country is mountainous in some parts and very pretty. Farther out are large "stations," and the fruit growing districts.

I made my bow, and retired from the Children's Hospital to give place to a resident medical officer, who supplied a want long felt.

Shortly after I went up the country again to a German settlement to nurse a case; it was like being in Germany, I fancy, for the people were all Germans, as well as the store-keeper; only the banks employed Englishmen. It was a very pleasant experience, everyone was so kind; I quite enjoyed the novelty.

(*To be continued.*)

## Figures and Tables

**Figure f1:**